# Oral Glucose Tolerance Test for the Screening of Glucose Intolerance Long Term Post‐Heart Transplantation

**DOI:** 10.3389/ti.2022.10113

**Published:** 2022-04-19

**Authors:** Stefan Roest, Marleen M. Goedendorp-Sluimer, Julia J. Köbben, Alina A. Constantinescu, Yannick J. H. J. Taverne, Felix Zijlstra, Adrienne A. M. Zandbergen, Olivier C. Manintveld

**Affiliations:** ^1^ Department of Cardiology, Thorax Center, Erasmus MC, University Medical Center Rotterdam, Rotterdam, Netherlands; ^2^ Erasmus MC Transplant Institute, Erasmus MC, University Medical Center Rotterdam, Rotterdam, Netherlands; ^3^ Department of Internal Medicine, Erasmus MC, University Medical Center Rotterdam, Rotterdam, Netherlands; ^4^ Department of Cardiothoracic Surgery, Thorax Center, Erasmus MC, University Medical Center Rotterdam, Rotterdam, Netherlands

**Keywords:** heart transplantation, comorbidity, oral glucose tolerance test, prediabetes, post-transplant diabetes mellitus

## Abstract

Post-transplant diabetes mellitus (PTDM) is a frequent complication post-heart transplantation (HT), however long-term prevalence studies are missing. The aim of this study was to determine the prevalence and determinants of PTDM as well as prediabetes long-term post-HT using oral glucose tolerance tests (OGTT). Also, the additional value of OGTT compared to fasting glucose and glycated hemoglobin (HbA1c) was investigated. All patients > 1 year post-HT seen at the outpatient clinic between August 2018 and April 2021 were screened with an OGTT. Patients with known diabetes, an active infection/rejection/malignancy or patients unwilling or unable to undergo OGTT were excluded. In total, 263 patients were screened, 108 were excluded. The included 155 patients had a median age of 54.3 [42.2–64.3] years, and 63 (41%) were female. Median time since HT was 8.5 [4.8–14.5] years. Overall, 51 (33%) had a normal range, 85 (55%) had a prediabetes range and 19 (12%) had a PTDM range test. OGTT identified prediabetes and PTDM in more patients (18% and 50%, respectively), than fasting glucose levels and HbA1c. Age at HT (OR 1.03 (1.00–1.06), *p* = 0.044) was a significant determinant of an abnormal OGTT. Prediabetes as well as PTDM are frequently seen long-term post-HT. OGTT is the preferred screening method.

## Graphical Abstract

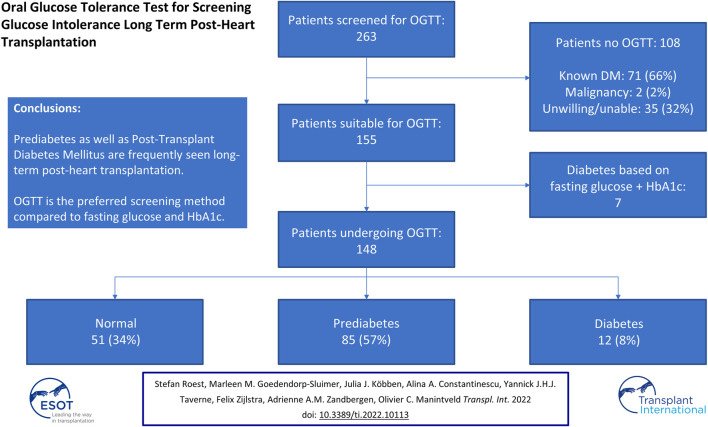

## Introduction

Type 2 diabetes mellitus is an increasing problem worldwide, leading to reduced life expectancy and increased risk for cardiovascular complications (1–3). Prediabetes is an intermediate metabolic state between normal glucose tolerance and diabetes mellitus, with a growing prevalence worldwide as well (over 45% when aged 65 years or more) (4, 5). Patients with prediabetes have an increased risk (of up to 70%) of developing type 2 diabetes mellitus; both leading to an increased risk of cardiovascular morbidity and mortality, in the general population as well as in patients with manifest atherosclerotic disease (6–9).

In solid-organ transplant recipients, the incidences of post-transplant diabetes mellitus (PTDM) are high, varying between 10 and 40% depending on the transplanted organ and definitions used (10–15, 17). Risk factors for the development of PTDM include general cardiovascular risk factors (such as body mass index, age and family history) as well as transplant-related causes (such as immunosuppressive regimen, viral infections) (10). The incidence of PTDM 5 years post-heart transplantation (HT) reported by the registry of the International Society for Heart and Lung Transplantation (ISHLT) is 34% (16). Unfortunately, information after 5 years is lacking in this registry. Currently, no other studies have been performed determining the prevalence of PTDM after 5 years in HT recipients.

Recently, data from the ISHLT registry showed that PTDM was associated with an increased risk for severe renal dysfunction, retransplantation and death (17). This warrants an early recognition of the disease in order to modify risk factors to decrease the risk for these worse outcomes. Moreover, in HT recipients, only one small study has been published that demonstrated no difference in outcomes between patients with prediabetes and diabetes in the year pre-HT (18). Studies on prevalence and outcomes of prediabetes post-HT are missing.

Previous studies in the general population have shown the added value of the oral glucose tolerance test (OGTT) to fasting glucose levels and glycated hemoglobin (HbA1c) for identifying patients with (pre)diabetes, with a significant number of patients being missed without the OGTT (19, 20). Therefore, in transplant patients, adding an OGTT is preferred in patients who are stable on immunosuppressive regimen to make a diagnosis of (pre)diabetes according to the American Diabetes Association (ADA) guidelines (4). In the guidelines of the ISHLT it is advised to periodically screen for PTDM after HT by fasting glucose levels or OGTT and HbA1c levels (21). However, studies on the added value of an OGTT compared to fasting glucose and HbA1c in HT recipients are missing.

In the current study, we investigated the prevalence of prediabetes and diabetes mellitus long-term post-HT and determinants for an abnormal OGTT long-term post-HT. Additionally, the added value of an OGTT compared to a fasting glucose and HbA1c is investigated.

## Patients and Methods

### Study Population

In this cross-sectional study, all adult HT patients who were more than 1 year post-HT that were seen at our outpatient clinic between August 2018 and April 2021 were screened to undergo an OGTT. Patients with known diabetes, an active infection/rejection treatment, patients who were treated for a malignancy and patients unwilling or unable to undergo OGTT were excluded. Information on immunosuppressive regimen has been published before (22). This study was conducted according to the Declaration of Helsinki and was approved by the Erasmus MC Ethics committee (MEC-2017-421).

### Oral Glucose Tolerance Test

The OGTT was performed according to the guidelines of de American Diabetes Association (ADA) (4). A fasting glucose level and HbA1c were measured at the outpatient clinic after which 75 g of glucose in a 200 ml solution was administered. After 2 hours a second glucose measurement was performed. Glucose measurements were performed using a finger prick (Accu-Check ^®^ Inform II, Roche) while HbA1c was measured in normal blood draws. In case a patient was unable to undergo an OGTT at the outpatient clinic due to the travel distance to the hospital, the OGTT was performed by the patient’s general practitioner with strict instructions.

#### Definitions

Transient hyperglycemia was defined as a patient needing insulin because of hyperglycemia in the first days post-operatively up until 45 days, based on the consensus document published by Sharif et al. (23). Patients still needing glucose-lowering drugs (oral or subcutaneous) after 45 days post-HT or developing PTDM during follow-up but who became normoglycemic without any glucose-lowering drugs during follow-up were labeled as recovered PTDM, as described in earlier studies (11). Cytomegalovirus (CMV) infection was defined as a patient with symptoms that were related to CMV replication.

The definitions for the results of the OGTTs were according to the ADA guidelines (4), which are demonstrated in Supplementary Table S1. When a patient had a test result in the diabetic range, a confirmation test was repeated after 6 weeks. When a patient had both an increased HbA1c level within the diabetes range as well as an increased fasting glucose within the diabetes range at the outpatient clinic, diabetes was diagnosed.

### Statistical Analysis

Normality of distribution was tested using the Shapiro-Wilks test. Continuous variables were expressed with a mean ± standard deviation (SD) when normally distributed and compared with a student t-test or one-way ANOVA depending on the number of groups. If the data were not normally distributed a median was presented with the 25th—75th percentile (Interquartile range, IQR) and compared using a Mann-Whitney or Kruskal Wallis test. Categorical variables were demonstrated as numbers with percentages (%) and compared with a Chi square or Fisher’s exact test where appropriate. To determine risk factors for an abnormal test result from OGTT (PTDM and prediabetes range tests) a binary logistic regression analysis was performed. First, an univariable analysis was performed including the sex of the recipient and the recipient age at the time of the HT. In order to not overfit the analysis, a propensity score was created which included variables that have been linked to PTDM in the literature. These variables included: ethnicity, time between heart transplantation and OGTT, heart failure etiology, transient hyperglycemia, resolved PTDM, number of rejections (patients with more than 3 rejections included into one group), cytomegalovirus disease, body mass index at time of OGTT, tacrolimus use at time of OGTT, and prednisolone use at time of OGTT. In a multivariable analysis, age, recipient sex and the propensity score were included. Additionally, an ordinal regression analysis was performed. In this analysis, age and a propensity score including all previously mentioned variables and recipient gender were included. A p-value < 0.05 was considered as statistically significant. The data were analyzed with IBM SPSS statistics 25 (IBM Corp., New Orchard Road, Amonk, NY10504, United States).

## Results

### Study Population

**FIGURE 1:**
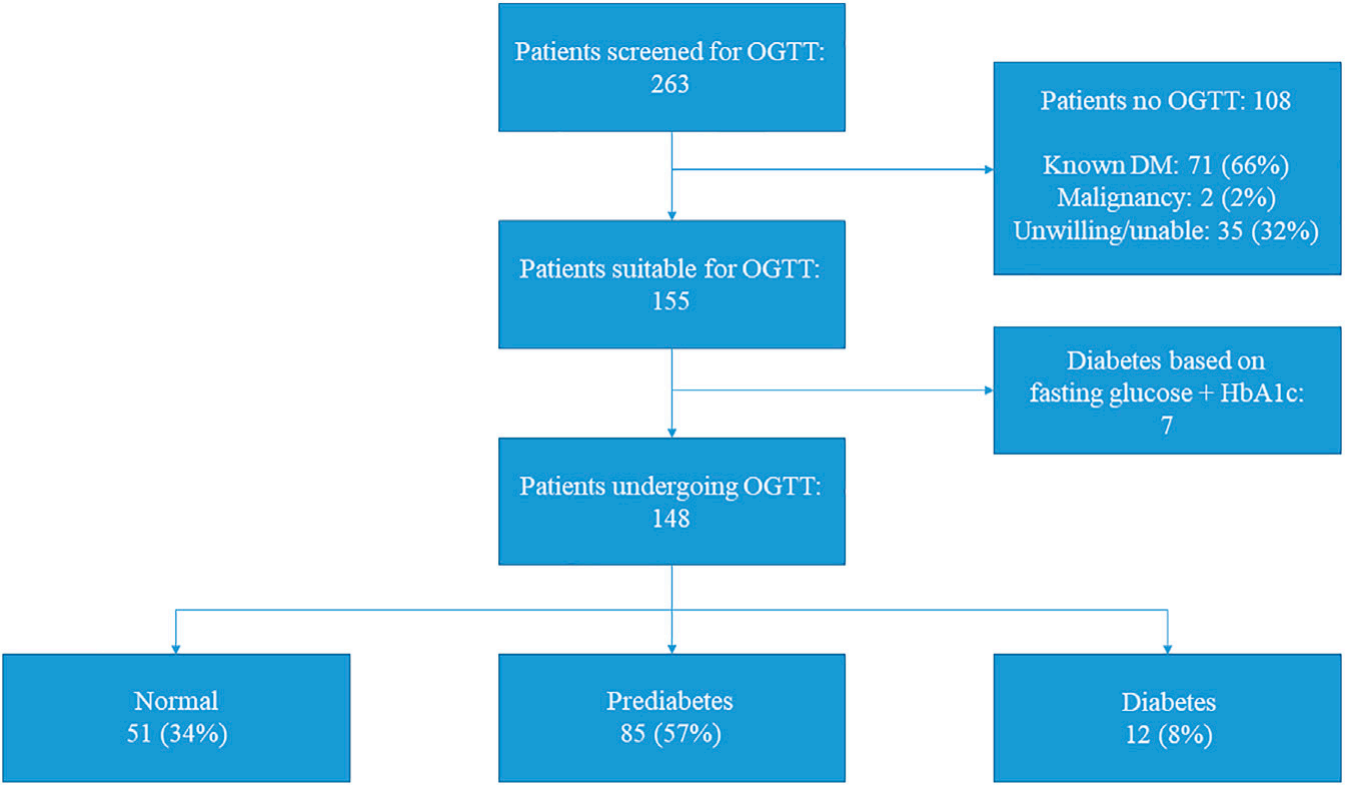
Results screening and oral glucose tolerance test. Results of screening and oral glucose tolerance test for all patients (*n* = 263). Of the 35 patients unwilling/unable to undergo oral glucose tolerance test, six (17%) died before a test could be performed. Abbreviations: DM, diabetes mellitus; HbA1c, glycated hemoglobin; OGTT, oral glucose tolerance test.

In total, 263 patients were screened at the outpatient clinic of whom 96 (37%) were female. The median age at HT was 47.1 [32.5–55.0] years old. The etiology of heart failure pre-HT was ischemic cardiomyopathy in 27% of patients; 17% had a left ventricular assist device pre-HT. During the admission directly after HT, 115 (44%) developed transient hyperglycemia. Reversed PTDM was seen in 37 (14%) patients, while 71 (27%) patients had known diabetes mellitus. Other baseline characteristics are demonstrated in Table 1. Of the 263 patients, 108 were excluded for OGTT after screening (Figure 1) based on the following exclusion criteria: known diabetes mellitus in 71 (66%) patients (of whom 20% developed diabetes pre-HT and 45% post-HT), 35 (32%) patients who were unable or unwilling to undergo OGTT (of whom six died before an OGTT was performed), and two (2%) patients underwent treatment for an active malignancy.

**TABLE 1:** Baseline characteristics all screened patients and divided into groups (patient who underwent oral glucose tolerance test versus those who did not).

Parameters	Whole Cohort	Patient Not Undergoing OGTT	Patients Undergoing OGTT	p-value
Number of patients	263	108	155	
Female	96 (37)	33 (31)	63 (41)	0.10
Ethnicity				0.25
Caucasian	227 (86)	89 (82)	138 (89)	
Black	8 (3)	5 (5)	3 (2)	
Other	28 (11)	14 (13)	14 (9)	
Age at HT (years)	47.1 [32.5–55.0]	48.7 [38.1–56.8]	46.2 [26.1–53.6]	0.047
Age at OGTT (years)			54.3 [42.2–64.3]	
Time HT—OGTT (years)			8.5 [4.8–14.5]	
Etiology heart failure				0.001
Ischemic CMP	70 (27)	40 (37)	30 (19)	
Non-ischemic CMP	193 (73)	68 (63)	125 (81)	
LVAD pre-HT	45 (17)	11 (10)	34 (22)	0.01
BMI at HT	23.1 ± 4.5	24.0 ± 4.9	22.4 ± 4.1	0.006
BMI at OGTT			25.8 [23.7–27.7]	
Prednisolone use 1 year post-HT	236 (90)	102 (94)	134 (87)	0.03
Medication at OGTT				
Tacrolimus			140 (90)	
Ciclosporin			15 (10)	
Mycophenolate mofetil			65 (42)	
Everolimus			27 (17)	
Prednisolone			93 (60)	
Prednisolone dosage (mg)			5.0 [5.0–7.5]	
Glycemic status				
Transient hyperglycemia post-HT	115 (44)	31 (29)	84 (54)	<0.001
Reversed PTDM post-HT	37 (14)	7 (6)	30 (19)	0.003
Known DM at OGTT	71 (27)	71 (66)	0 (0)	<0.001
DM pre-HT	22 (8)	22 (20)	0 (0)	
DM post-HT	49 (19)	49 (45)	0 (0)	
Rejections^a^	1 [0–2]	1 [0–2]	1 [0–2]	0.007
CMV infection	53 (20)	19 (18)	34 (22)	0.39

a Number of rejections treated with methylprednisolone.

Baseline characteristics of all patients and those who underwent oral glucose tolerance test (including patients in whom the diagnosis was determined based on fasting glucose and HbA1c). Continuous variables are demonstrated with mean ± standard deviation when normally distributed and median with [25th—75th percentile] when not normally distributed. Categorical variables are demonstrated with numbers and (%).

Abbreviations: BMI, body mass index; CMP, cardiomyopathy; CMV, cytomegalovirus; DM, diabetes mellitus; HT, heart transplantation; LVAD, left ventricular assist device; OGTT, oral glucose tolerance test.

### Oral Glucose Tolerance Test

In 148 out of 155 patients an OGTT was performed since in seven patients PTDM could be diagnosed based on solely the fasting glucose level in combination with the HbA1c (Figure 1). Baseline characteristics are demonstrated in Table 1. Forty-one percent were female and the most common ethnicity was Caucasian (89%). The median age at the time of the OGTT was 54.3 [42.2–64.3] years old and patients were 8.5 [4.8–14.5] years post-HT. Median BMI was 25.8 [23.7–27.7] kg/m^2^ and transient hyperglycemia and reversed PTDM were seen in 54% and 19%, respectively.

When including the results after 6 weeks to confirm (when needed) the diagnosis of PTDM, based on OGTT 51 (34%) of patients had a normal glucose tolerance, 85 (57%) had a prediabetes range test and 12 (8%) had a PTDM range test. Together with the 7 patients in whom the diagnosis was confirmed using fasting glucose and HbA1c, 51/155 (33%) had a normal range test, 85/155 (55%) had a prediabetes range test and 19/155 (12%) of patients had a diabetes range test during the study period. The baseline characteristics of the patients undergoing OGTT (*n* = 155) stratified by OGTT result are demonstrated in Table 2.

**TABLE 2:** Baseline characteristics based on oral glucose tolerance test result.

	Results OGTT			
Parameters	Normal Range	Prediabetes	PTDM	p-value
Number of patients	51	85	19	
Female	26 (51)	30 (35)	7 (37)	0.18
Ethnicity				0.28
Caucasian	47 (92)	73 (86)	18 (95)	
Black	2 (4)	1 (1)	0 (0)	
Other	2 (4)	11 (13)	1 (5)	
Age at HT (years)	38.7 [22.2–52.1]	49.3 [33.9–55.1]	45.6 [25.4–50.8]	0.08
Age at OGTT (years)	49.7 [30.4–56.7]	57.7 [46.9–66.1]	54.8 [42.0–65.4]	0.019
Time HT—OGTT (years)	8.4 [3.5–11.8]	8.5 [5.1–15.0]	11.5 [5.2–19.1]	0.13
Etiology heart failure				0.23
Ischemic CMP	6 (12)	19 (22)	5 (26)	
Non-ischemic CMP	45 (88)	66 (78)	14 (74)	
LVAD pre-HT	12 (24)	18 (21)	3 (16)	0.78
BMI at HT	22.3 ± 4.7	22.4 ± 3.6	22.9 ± 4.4	0.85
BMI at OGTT	25.1 [23.3–27.1]	25.9 [24.1–27.6]	26.7 [23.0–30.6]	0.56
Prednisolone use 1 year post-HT	42 (82)	75 (88)	17 (89)	0.74
Medication at OGTT				
Tacrolimus	49 (96)	75 (88)	16 (84)	0.21
Ciclosporin	2 (4)	10 (12)	3 (16)	0.21
Mycophenolate mofetil	23 (45)	35 (41)	7 (37)	0.81
Everolimus	6 (12)	19 (22)	2 (11)	0.20
Prednisolone	27 (53)	50 (59)	16 (84)	0.056
Prednisolone dosage (mg)	5 [5–7.5]	5 [5–7.5]	7.5 [5–10]	0.20
Glycemic status				
Transient hyperglycemia post-HT	32 (63)	46 (54)	6 (32)	0.054
Reversed PTDM	6 (12)	17 (20)	7 (37)	0.07
Rejections^a^	1 [0–1]	1 [0–2]	1 [0–2]	0.18
CMV infection	10 (20)	20 (24)	4 (21)	0.86

a Number of rejections treated with methylprednisolone.

Baseline characteristics of all patients and those who underwent oral glucose tolerance test (including patients in whom the diagnosis was determined based on fasting glucose and HbA1c). Continuous variables are demonstrated with mean ± standard deviation when normally distributed and median with [25th–75th percentile] when not normally distributed. Categorical variables are demonstrated with numbers and (%).

Abbreviations: BMI, body mass index; CMP, cardiomyopathy; CMV, cytomegalovirus; DM, diabetes mellitus; HT, heart transplantation; LVAD, left ventricular assist device; OGTT, oral glucose tolerance test; PTDM, post-transplant diabetes mellitus.

In total, 40 patients (27%) were within 5 years post-HT and 108 (73%) were more than 5 years post-HT. When the results of the OGTTs were stratified according to the time post-transplant, no significant differences were seen in the results (*p* = 0.33) as is demonstrated in Supplementary Table S2.

### Additional Value of Oral Glucose Tolerance Test

**FIGURE 2:**
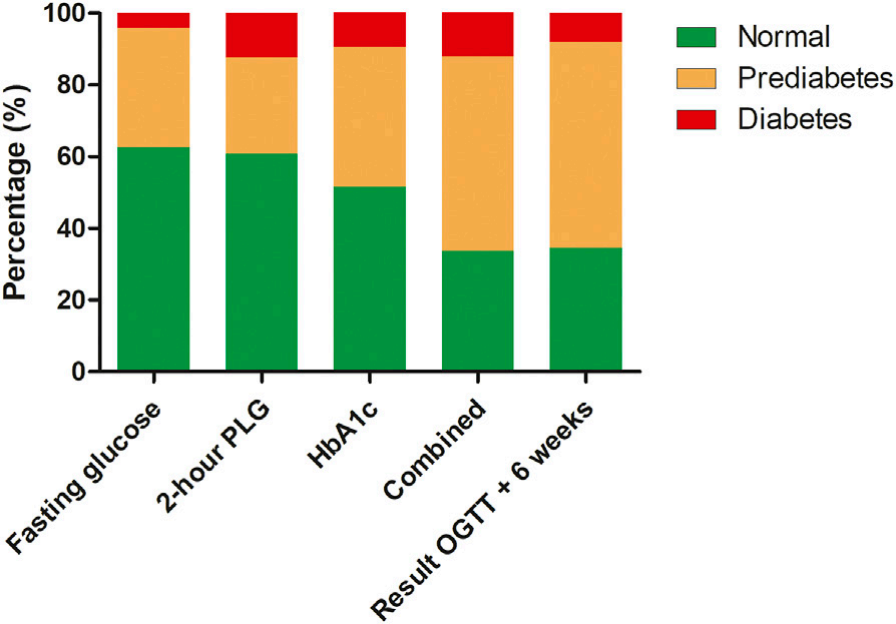
Results of oral glucose tolerance test specified by detection method, combination of all the results and with confirmation test after 6 weeks. Abbreviations: HbA1c, glycated hemoglobin; OGTT, oral glucose tolerance test; PLG, postload glucose.

For the patients who underwent an OGTT, the results of each component of the OGTT (fasting glucose, 2 h postload glucose, and HbA1c) are demonstrated in Figure 2. Based on the OGTT data, 123 (83%) of the patients with (a combination of) only a fasting serum glucose test and an HbA1c measurement would have been be correctly classified. Of the remaining 25 (17%) patients who were not correctly classified, 14 (56%) had prediabetes and 11 (44%) diabetes. When including the results of the repeated OGTT after 6 weeks (confirming the PTDM diagnosis), 127 (86%) would have been classified correctly, while 21 (14%) would not. Of the patients who were not correctly classified, 15 (71%) had prediabetes and 6 (29%) PTDM. When looking at all patients with prediabetes range results, 15 (18%) of patients would have been missed without an OGTT. In diabetes range patients this would have been the case in 6 (50%) of the cases.

### Determinants of Abnormal Oral Glucose Tolerance Test

In order to define determinants for an abnormal OGTT, patients with a prediabetes and PTDM range test were combined. Univariable analysis demonstrated that age at HT (OR 1.02 (1.00–1.04), *p* = 0.036) was a significant determinant of an abnormal test, while recipient gender was not (OR 0.53 (0.27–1.05), *p* = 0.07) (Table 3). When both recipient age, recipient gender and the propensity score were included in one model, age at HT was still a significant determinant (OR 1.03 (1.00–1.06), *p* = 0.044). In ordinal regression analysis, this association between recipient age at HT and OGTT outcome was confirmed (OR 1.03 (1.00–1.05), *p* = 0.044) (Table 4).

**TABLE 3:** Logistic regression analysis investigating determinants of an abnormal oral glucose tolerance test result (prediabetes or diabetes range test).

	Abnormal OGTT Result		
	OR (95% CI)		
	Univariable	Model 1	Model 2
Age recipient at HT	1.02 (1.00–1.04)	1.02 (1.00–1.04)	1.03 (1.00–1.06)
P-value	0.036	0.07	0.044
Female recipient	0.53 (0.27–1.05)	0.59 (0.29–1.18)	0.60 (0.28–1.31)
P-value	0.07	0.14	0.20

Univariable analysis of recipient sex and recipient age at heart transplantation individually in the model.

Model 1: Model including sex and age at the time of the heart transplantation of the recipient.

Model 2: Model including sex and age adjusted for the propensity score which included the following parameters: ethnicity, time between heart transplantation and CT scan, heart failure etiology, transient hyperglycemia, resolved PTDM, number of rejections*, cytomegalovirus disease, body mass index at time of OGTT, tacrolimus use at time of OGTT, and prednisolone use at time of OGTT. *Patients with 3 or more rejections were combined into one group due to the small number of patients.

Abbreviations: CI, confidence interval; HT, heart transplantation; OGTT, oral glucose tolerance test; OR, odds ratio.

**TABLE 4:** Ordinal regression analysis investigating determinants of the oral glucose tolerance test results.

	Abnormal OGTT result	
	OR (95% CI)	
	Model 1	Model 2
Age recipient at HT	1.02 (1.00–1.04)	1.03 (1.00–1.05)
P-value	0.023	0.044

Model 1: unadjusted

Model 2: Model including age adjusted for the propensity score which included the following parameters: recipient sex, ethnicity, time between heart transplantation and CT scan, heart failure etiology, transient hyperglycemia, resolved PTDM, number of rejections*, cytomegalovirus disease, body mass index at time of OGTT, tacrolimus use at time of OGTT, and prednisolone use at time of OGTT.

*Patients with 3 or more rejections were combined into one group due to the small number of patients. Abbreviations: CI, confidence interval; HT, heart transplantation; OGTT, oral glucose tolerance test; OR, odds ratio.

## Discussion

This study shows that impaired glucose metabolism is highly prevalent in patients long term post-HT. Based on OGTT, 104 of 155 patients without known PTDM (67%) had an abnormal test of whom 85 (55%) had a prediabetes range test, while 19 (12%) had a PTDM range test. When stratified by time since HT (≤ or > 5 years), there was no difference in the test results (*p* = 0.33). An OGTT demonstrated 18% more prediabetes and 50% more PTDM compared to a fasting glucose level and HbA1c only. Age at HT was significantly associated with the result of the OGTT.

This is the first study investigating the long-term prevalence of prediabetes and diabetes in HT recipients. In the registry from the ISHLT, diabetes mellitus is monitored up until 5 years post-HT, where diabetes status is reported in accordance with the clinical diagnostic guidance in place at the reporting transplant center (16). The incidence of diabetes mellitus at 5 years in the registry between 1995 and 2017 was 33.8%, which is higher than what was seen in our population before we performed the OGTT (27%) (16). Even though our patients were regularly seen at the outpatient clinic, 12% of patients had a PTDM range test when using the OGTT. Overall, this increased the total number of patients with PTDM from 27% to 34%, a relative increase of 26%. This demonstrates that a high percentage of patients have unnoticed PTDM despite regular blood test for abnormal glucose metabolism (mostly by assessment of non-fasting glucose levels). This is emphasized by our analysis on the added value of the OGTT compared to a fasting glucose and HbA1c. By performing an OGTT, prediabetes and PTDM were diagnosed in 18% and 50% more patients, respectively. To our knowledge, this is the first study demonstrating this significant advantage in heart transplantation recipients. Ussif et al. investigated kidney transplant recipients who underwent OGTT 1 year post-transplant (24). In this study, an OGTT only identified two patients who would have been missed compared to a combination of fasting glucose and HbA1c (24). This could be due to the difference in the timing of the OGTT, or a significant change in metabolism (given the other comorbidities that patients develop over time) in heart transplant recipients (16). However, our study illustrates that regular testing of the glycemic status through OGTT is warranted in (heart) transplant patients as is recommended by the American Diabetes Association (4).

In our study, a total of 55% of patients had a prediabetes range test. It is essential to monitor the glucose metabolism closely in this patient population because of their high cardiovascular risk profile and highly prevalent risk factors such as hypertension, dyslipidemia, renal dysfunction but also the chronic use of steroids (25). When these patients also have PTDM, the cardiovascular risk significantly further increases (17). Whether this is also applicable in HT patients with prediabetes needs to be further investigated. Studies in non-transplant patients have demonstrated that individuals with prediabetes have a significantly increased risk to develop diabetes mellitus as well as cardiovascular morbidity and mortality (6, 7). Moreover, in a recent study in kidney transplant recipients, patients with prediabetes had similar risks for cardiovascular mortality as patients with PTDM (26). This demonstrates that patients with prediabetes should be monitored carefully and diagnosed early. As impaired glucose metabolism is a modifiable risk factor, the question remains whether an intervention will result in the reduction of cardiovascular events. Unfortunately, currently, no studies have been performed in HT recipients. Only one study showed that patients with prediabetes pre-HT had no increased risk of mortality after a median of 50 months post-HT (18). This study only used HbA1c levels to determine whether a patient had prediabetes, which could underestimate the true number of patients with prediabetes. Overall, the question remains whether patients who develop prediabetes post-HT have an accelerated progression of cardiac allograft vasculopathy (CAV) and/or an increased risk of micro- or macrovascular complications and mortality.

Age at HT was a significant determinant for an abnormal OGTT. Unfortunately, we were not able to include other determinants of PTDM in the analysis due to the relatively small study population, such as immunosuppressive regimen (prednisolone, tacrolimus), rejections, and CMV infection (10, 17). However, in univariable analysis, these factors were not associated with worse outcomes. There are several reasons for this. First of all, in patients who are more than 1 year post-HT, the significance of risk factors such as rejections probably become less important in the development of PTDM, especially since most rejections occur in the first year post-HT (16) and steroid use is tapered after the first year as is seen in our study. In univariable analysis, prednisolone use during OGTT was not associated with the OGTT outcome. Even though prednisolone is a risk factor for PTDM (10), one study in kidney transplant recipients demonstrated that patients who use prednisolone in a daily dosage of 5 mg or lower had no improvement in insulin sensitivity (assessed with glucose clamp technique) (27). This was confirmed by a Cochrane review including kidney transplant recipients which demonstrated that withdrawal of steroids did not decrease the risk on PTDM (28). Moreover, withdrawal of prednisolone increased the risk for acute rejection (28). In our opinion, prednisolone withdrawal should only be performed in patients who have a low risk for the development of an acute rejection episode and for other reasons than PTDM prevention. In other patients, steroids could be used with a daily dosage of 5 mg in order to reduce PTDM risk as much as possible. When patients do develop diabetes, the newer drugs to treat diabetes are of special interest since these drugs have cardio-renal-metabolic effects (i.e., sodium-glucose cotransporter-2 (SGLT2) inhibitors or glucagon-like peptide 1 (GLP1) analogues) (29,30).

Our study has several limitations. First of all, this is a single-center study which comes with all its limitations such as generalizability. In our study population, around 10% of patients were unable or unwilling to undergo OGTT which could increase the risk for selection bias. Ultimately, this could mean that the numbers in our study underestimate the frequency of prediabetes and PTDM long-term post-HT. Furthermore, most patients in our population were Caucasian which makes it difficult to extrapolate our study results to populations consisting of other ethnicities.

In conclusion, our study demonstrated that both prediabetes and PTDM are frequently observed in patients not known with PTDM long-term post-HT. Age at HT was a determinant for an abnormal OGTT. OGTT is the preferred test to screen for prediabetes and PTDM since it identifies significantly more patients than (fasting) glucose and HbA1c levels alone. Future studies are needed to investigate the impact of prediabetes and PTDM diagnosed long-term post-HT on transplant-related outcomes as well as future cardiovascular complications in this high-risk population. Furthermore, studies are needed to investigate the effects of glucose-lowering interventions (with lifestyle and/or medication) on progression of prediabetes to PTDM and prevention of (cardiovascular) complications.

## Data Availability

The datasets presented in this article are not readily available due to privacy reasons. Reasonable requests to access the datasets should be directed to o.manintveld@erasmusmc.nl.

## Abbreviations

ADA, American Diabetes Association; BMI, body mass index; CAV, cardiac allograft vasculopathy; CMV, cytomegalovirus; HbA1c, glycated hemoglobin; HT, heart transplantation; ISHLT, International Society for Heart and Lung Transplantation; LVAD, left ventricular assist device; OGTT, oral glucose tolerance test; OR, odds ratio; PTDM, post-transplantation diabetes mellitus.
