# Supplementary material for: Oral Glucose Tolerance Test for the Screening of Glucose Intolerance Long Term Post‐Heart Transplantation
Source: Transpl Int. 2022 Apr 19;35:10113. doi: 10.3389/ti.2022.10113 (PMC9061939; doi:10.3389/ti.2022.10113)
Supplement: Supplementary file 1 [file DataSheet1.docx]

Supporting Information of manuscript: “Oral Glucose Tolerance Test for the Screening of Glucose Intolerance Long Term Post-Heart Transplantation”.

**Supplemental Table S1.**

| **Diagnosis** | **Fasting Glucose** | **Glucose after two hours** | **HbA1c** |
| --- | --- | --- | --- |
| Diabetes Mellitus | ≥ 126 mg/dL  (≥ 7.0 mmol/L) | ≥ 200 mg/dL  (≥ 11.1 mmol/L) | ≥ 6.5 %  (≥ 48 mmol/mol) |
| Prediabetes | 100 – 125 mg/dL  (5.6 – 6.9 mmol/L) | 140 – 199 mg/dL  (7.8-11.0 mmol/L) | 5.7 – 6.4 %  (39 – 47 mmol/mol) |
| Normal | < 100 mg/dL  (< 5.6 mmol/L) | < 140 mg/dL  (< 7.8 mmol/L) | < 5.7 %  (< 39 mmol/mol) |

Definitions used during oral glucose tolerance test according to the American Diabetes Association guidelines [4]. Definitions given in both European and American measurements.

**Supplementary Table S2.**

**Results oral glucose tolerance test stratified by time since heart transplant**

| Parameters | All | ≤ 5 years since HT | > 5 years since HT |
| --- | --- | --- | --- |
| Number of patients | 148 | 40 | 108 |
| Normal range | 51 (34) | 17 (43) | 34 (31) |
| Prediabetes range | 85 (57) | 19 (48) | 66 (61) |
| PTDM range | 12 (8) | 4 (10) | 8 (7) |

Results oral glucose tolerance test (OGTT) divided into patients who had an OGTT within 5 years and those who had an OGTT after 5 years. Numbers given with (%), p=0.33.

Abbreviations: HT, heart transplantation; PTDM, post-transplant diabetes mellitus.
